# A Case-Controlled Comparison of Behavioural Arousal Levels in Urine Spraying and Latrining Cats

**DOI:** 10.3390/ani10010117

**Published:** 2020-01-10

**Authors:** Daniela Ramos, Archivaldo Reche-Junior, Priscila Luzia Fragoso, Rupert Palme, Patricia Handa, Marie Odile Chelini, Daniel Simon Mills

**Affiliations:** 1 Psicovet Canine and Feline Behaviour and Welfare Center, Rua Inhambu 1080, São Paulo 04520-013, Brazil; 2 Department of Medical Clinics, Faculty of Veterinary Medicine and Animal Science, University of São Paulo, São Paulo 05508-270, Brazil; valdorec@usp.br (A.R.-J.); prifragoso@yahoo.com.br (P.L.F.); 3 Department of Biomedical Sciences, University of Veterinary Medicine, Veterinärplatz 1, 1210 Vienna, Austria; Rupert.Palme@vetmeduni.ac.at; 4 Department Experimental Psychology, Psychology Institute, University of São Paulo, São Paulo 05508-030, Brazil; handa.p.atih@gmail.com (P.H.); marodiche@gmail.com (M.O.C.); 5 School of Life Sciences, College of Sciences, University of Lincoln, Lincoln LN6 7DL, UK; dmills@lincoln.ac.uk

**Keywords:** feline, housesoiling, stress, marking behaviour

## Abstract

**Simple Summary:**

Urination outside the litterbox (also known as periuria) is a very frequent problem seen by veterinary behaviourists and is a common reason for the relinquishment of cats. Veterinary behaviour textbooks describe two forms of periuria (spraying and latrining), including characteristics of both, and speculations, such as spraying is more closely associated with stress. With the aim of evaluating the arousal underpinning emotional stress in cats showing periuria, we studied recorded behaviours as well as faecal cortisol metabolite (FCM) levels of 11 “sprayer” and 12 “latriner” cats along with their controls (i.e., cats that did not show periuria) from the same multi-cat homes of three to nine cats. The results indicated that households in which a cat exhibits urine spraying are generally more aroused than households with latrining cats, but “sprayers” are not more aroused than their housemates. In practical terms, such results suggest that behaviour management to control periuria in these households should be focused on all cats not just the “sprayers”.

**Abstract:**

It is often suggested that both latrining and spraying in the home are associated with increased stress in cats. However, the scientific evidence for this is weak. We therefore examined faecal cortisol metabolite (FCM) levels in subjects using a case-control design. Eleven spraying and 12 problematic latrining cats (assessed as healthy after detailed medical examinations on an initial population of 18 spraying and 23 latrining cats) were assessed along with behaviourally normal and similarly healthy control subjects from the same multi-cat (n = 3–9) households. Individual faecal samples were collected by owners from both “case” and “control” cats after observing them defecate in all but one pair in each group. A total of five samples per cat (typically taken on a weekly basis) were collected and submitted to extraction procedures prior to FCM analysis via an 11-oxoaetiocholanolone enzyme immunoassay (EIA). Participant cats, both “cases” (nine “sprayers” and eight “latriners”) and controls, were also individually video recorded (together with the owner) for 5 min in a dedicated room. FCM levels were significantly higher in individuals (“sprayers” and their controls) from spraying households than from the latrining households (“latriners” and their controls), but there was no significant difference between cats from the same household. Within a video observation test, cats from spraying houses spent proportionally more time moving (as opposed to stationary), but again there was no difference between cats from the same house. These results indicate that households in which a cat exhibits urine spraying, are generally more aroused, but “sprayers” are not more aroused than their housemates. Accordingly, we suggest appropriate management needs to be applied to the whole household to help alleviate the potential stress of all the cats in the home, and not just the one expressing this through urinary spraying behaviour.

## 1. Introduction

Inappropriate urinary soiling of the house (periuria) in the form of marking (spraying) or micturition (latrining) behaviour is the most common problem seen by veterinary behaviourists and a common behavioural problem reported by owners [1], with an overall prevalence of 45% recorded across European behaviourists by the Association of Pet Behaviour Counsellors (APBC) [2]. In North America, the figures are 67% in the USA and 60% in Canada while the figures are 48% in Australia [3]. In South America, in Brazil, a prevalence of 26.4% was recorded within the feline behavioural caseload (n = 70) of two of the current authors from 2008 to 2014 [4].

In a clinical context, spraying has traditionally been distinguished from latrining on the basis of their form [5,6,7,8,9,10,11,12]. For instance, it is commonly suggested that spraying involves small quantities of urine being deposited on vertical surfaces (or on significant horizontal spots), with the cat typically in a standing posture and the tail held high [6,8,9]. “Sprayer” cats are generally believed to continue to use an appropriate latrine (e.g., litter box in the home) for both urination and defecation [6,8,9]. By contrast, latrining is most often characterized by urine being deposited on horizontal surfaces with the cat in a squatting posture [6,8,9]. “Latriner” cats frequently give up using the latrine for either or both urine and faeces deposition and so these may be found in inappropriate locations [6,8,9]. However, these assumptions may be unreliable [13], with Neilsen [14] reporting that the volume of urine eliminated during spraying is not significantly different to that produced during micturition.

Although spraying and latrining are distinguishable in terms of their functional basis, with the former commonly linked to sexual or territorial marking behaviours and the latter with latrine-related elimination [5,6,7,8,9,10,11,12], neither the presentation nor treatment may be as straightforward as commonly implied. For example, Pryor et al. [15] treatment regimen for spraying cats has a strong emphasis on litterbox cleanliness while Ramos et al. [16] found that while there was no difference in the prevalence of medical problems in “sprayer” and “latriner” cats, latrining houses (but not spraying houses) presented with more medical problems in their control cats. The relationship between each of these problems and the stress response also appears unclear, with it often assumed that cats who spray are doing so specifically because they are anxious over something in their environment [17,18] while those exhibiting latrining problems are simply finding an alternative outlet for their need for micturition, and so are not distressed to the same degree. However, to date, data to support or refute these hypotheses are either lacking, in the case of latrining, or inconsistent, in the case of spraying.

In the only previous study known to the authors that has examined the physiological stress response of urine spraying cats [19], the apparently higher average cortisol:creatinine ratio at 9:9 in the *spraying* cat population (compared to 5:9 in normal healthy cats [19], appears, on closer inspection, to be largely the result of an outlier, with a ratio of 96:7 in this population. When this exceptional individual is excluded, then the average cortisol:creatinine ratio among spraying cats falls to 6:92 [19]. Sickness might also raise cortisol levels [20], and it is possible that one subject had a preclinical illness. However, perhaps the most important issue with this latter study is the lack of a properly matched control population, such as subjects from within the same household. Accordingly, there is a need to critically appraise common assumptions and interpretations of the data made in relation to both the characterization and causes of urine spraying and latrining behaviour in domestic-owned cats.

The risk of urine spraying in a home appears to increase with the number of cats in the house [21]. Furthermore, both physical (e.g., the addition of new furniture) and social (e.g., a change in the owner’s schedule) environmental changes appear to be commonly associated with the onset urine spraying in cats [22]. These observations are consistent with urine marking behaviour being a response to threats to the core area of the cat’s normal territory.

Schwartz [23] and Bowen and Heath [10] propose that spraying is reassuring for the cat that displays it, with urine deposits increasing the sense of security, thus alleviating anxiety and distress. In this case, it might be predicted that the act of urine marking may actually lower the physiological stress response of subjects in a stressful environment.

While it may be assumed that non-housesoiling animals within the same home are normal, there has been no investigation of the physiological state of these subjects to determine whether this is the case. This is important, since if spraying is a coping response, the absence of spraying may indicate a lack of a need to spray or a failure to adopt a coping strategy. These competing explanations may only be differentiable by including an examination of the physiological state of non-soiling healthy subjects living within the same environment as housesoiling ones.

Therefore, this research, which was part of a larger study on feline periuria conducted in 2012 (with a previous piece focused on the medical problems being already published [16]), aimed to evaluate the differences in the levels of behavioural and physiological arousal in cats exhibiting either spraying or latrining behaviour versus control subjects from the same households, as well as the differences between non-housesoiling subjects from the two environments.

## 2. Materials and Methods

### 2.1. Recruitment and Selection

Research publicity aimed at selecting housesoiling cats and matched controls (without periuria) from the same multi-cat households (3–9 cats) was promoted in several ways. An information poster about feline housesoiling was posted at strategic points of the veterinary college at the University of São Paulo (FMVZ/USP) and in several veterinary clinics in Sao Paulo and neighbouring cities. It was also sent by email to a list of students of the veterinary college and posted on cat breeder websites and Internet communities related to pet cats. Owners were instructed to contact the main researcher by email or telephone for consideration for inclusion.

All suitable households were then visited by the main researcher, and from each of them, at least a dyad was selected to form a “case-control” study. This consisted of a cat displaying periuria (i.e., the “case” cat) and another cat, preferably of same sex and age as the “case” cat, described by the owner as never displaying periuria (i.e., “control” cat), with both neutered and apparently healthy. Cats displaying any other symptoms besides periuria, or any other overt behavioural problem, were considered to be unhealthy and thus were not selected for inclusion in either of the groups.

During visits, the owners were informed about the research objectives as well as its stages and a behavioural diagnosis was made. Following the “key” features proposed by Horwitz [6] for the behavioural diagnosis of periuria (i.e., urine amount and distribution outside the litterbox, type of surface in which urine is deposited inappropriately, cat´s posture during inappropriate urination, and litterbox use), which of the groups the pair would be allocated to was determined. In case of any ambiguity, a European specialist in veterinary behavioural medicine (DM) was consulted.

#### 2.1.1. Group S (“Sprayers”)

Twenty-one urine spraying “case-control” dyads were initially recruited. They were then subjected to a physical examination by a feline specialist (PF) followed by complementary exams (i.e., complete blood count, biochemical profile, urine exam and urine culture, ultrasound of the urinary system), which were all conducted at the veterinary hospital at the University of São Paulo (HOVET-USP), with a view to the selection of participant cats. In the case of three dyads, examinations could not be concluded for differing reasons (e.g., owner did not return for a second urine collection, which was required as the first one was not successful; cat performed spraying only around the litter box area) and therefore they were excluded from further involvement in the research.

Complete preliminary examinations were thus achieved with 18 urine spraying “case-control” dyads. As some individuals within these dyads presented medical problems, only 11 healthy urine spraying “case-control” dyads were finally selected to group S (the medical findings in these subjects were included in a separate publication [16]). Among the 11 “case” cats, there were two females and nine males, with all being neutered; 10 were mixed breeds and one was Persian. Their age ranged from 3 to 12 years old (SD = 2.44). “Control” cats were 6 females and 5 males, with all being neutered; 10 were of mixed breeds and one was Siamese. Their age ranged from 9 months to 14 years old (SD = 4.48). Households recruited for group S had, on average, 6 cats per household. Four of the 11 “case-control” dyads came from two households; there were therefore 9 (nine) different households in group S. These were all houses, with five of them having free outside access and three of them having an enclosed yard. In the remaining household, cats did not have any form of outside access.

#### 2.1.2. Group L (“Latriners”)

Thirty-four inappropriate latrining “case-control” dyads were initially recruited. As with group S, they were then submitted to a physical examination by a feline specialist (PF) followed by complementary exams, all conducted at the university veterinary hospital (HOVET-USP). For six dyads, the examinations could not be concluded for differing reasons (e.g., cat became sick; owner did not return for a second urine collection, which was required as the first one was not successful; cat was highly aggressive, thus not allowing physical examination accompanied by blood and urine collection) and therefore they were excluded from the research; a further five dyads were from households where other cats exhibited urine spraying and therefore they were not eligible for inclusion. Thus, 23 pairs were selected for initial inclusion in group L; all lived in households without the problem of urine spraying.

Within these dyads, in 11 instances, at least one of the cats presented medical problems [16]; this left 12 healthy “case-control” latrining dyads. Of these, 10 were females and two males, with all being neutered; six were mixed breeds, two Persians, two Maine Coons, and two Ragdolls. Their age ranged from 2 to 8 years (SD = 2.19). As for the “control” cats, they were 7 females and 5 males, with all being neutered; 9 were mixed breeds, 2 Maine Coons, and 1 Persian. Their age ranged from 6 months to 12 years (SD = 3.35). Households recruited for group L had, on average, 4.6 cats per household. Two of the 12 “case-control” dyads came from only one household; there were then 11 different households in group L. These were six houses and five flats. In five houses, there were enclosed yards, and in one of them, cats did not have any form of outside access. In all flats but one, there were balconies. Thus, there was no free outside access in any of the spraying households.

### 2.2. Faecal Samples and Determination of Cortisol Metabolites

During home visits, owners were informed about the process for the evaluation of feline stress via faecal cortisol metabolites (FCMs) and they were told about how to proceed with the faecal collections of both cats, “case” and “control”, which had to be completed before the medical examinations at the university veterinary hospital. Detailed instructions regarding the individual collection and the immediate storage in the owner’s freezer were given to them. Given the difficulties involved in the procedure, considering the existing number of cats in the household and the need to witness the defecation, a specific frequency of collection was not defined. It was stipulated that the collection should be on different days, one collection a week, with a maximum period of 50 days available for completion. The owners received 10 collection pots, as 5 samples per cat were to be collected. Samples were transported to the university in thermal boxes and were then transferred to polypropylene tubes and stored in a −80 °C freezer until analysis.

For analysis, samples were dried and weighted, and steroids were extracted and dried again for transportation at the Sao Paulo State University (UNESP, Jaboticabal). For these, 0.2 g aliquots of dry faeces were placed in glass vials (15 mL) to which 5 mL of 90% methanol were added. A multi vortex unit was used for a 15-min homogenization of the sample followed by 15 min of 3000 rpm centrifugation. Extracts were then dried for transportation in Eppendorf tubes. Faecal analyses were conducted at the University of Veterinary Medicine, Vienna (Austria) as a single batch. Re-suspension and dilution procedures were undertaken according to the methods used in that institution [24,25]. Samples were then analysed using an 11-oxoaetiocholanolone enzyme immunoassay (EIA), which measures a group of glucocorticoid metabolites largely excreted by domestic cats: 11,17 dioxoandrostanes. This method has been proven to be both valid and reliable [24,25].

For each cat, the median values of the five samples were then used for comparative analysis.

### 2.3. Recording of Cat Behaviour

Participant cats were all video recorded (Sony 19TRV) in a dedicated room during their visit to the veterinary hospital before medical examinations were performed. The aim of this was to characterize the cats’ behavioural responses to a stressful situation (i.e., a visit to the vet), even though all actions and procedures by the vets were conducted in a feline friendly manner. “Cases” and “controls” were alternated in a pre-defined randomization schedule as to who was first recorded. Five minute sessions were conducted independently for each of the cats, with only the owner and the cat present at the start of the session. In the first part of the session, the owner was instructed to place the cat on the floor as soon as the researcher left the room and the recording begun. The cat carrier was kept closed and placed in a designated location within the recording area. The cat was left free in the room for about three minutes. The owner was instructed to remain seated in the designated chair the whole time, without promoting any interaction with the cat.

After this time, the researcher entered the room and put a saucer containing wet cat food on the floor, near the chair where the owner was seated and then left the room. The food was offered as a further action to help infer the stress levels of the cats, with the rationale that if it was high, they would be unlikely to eat it. The second part of the session began when the owner placed the cat on the floor in front of the saucer. The same recommendations were given, and the cat remained free in the room for another two minutes.

**Figure 1:**
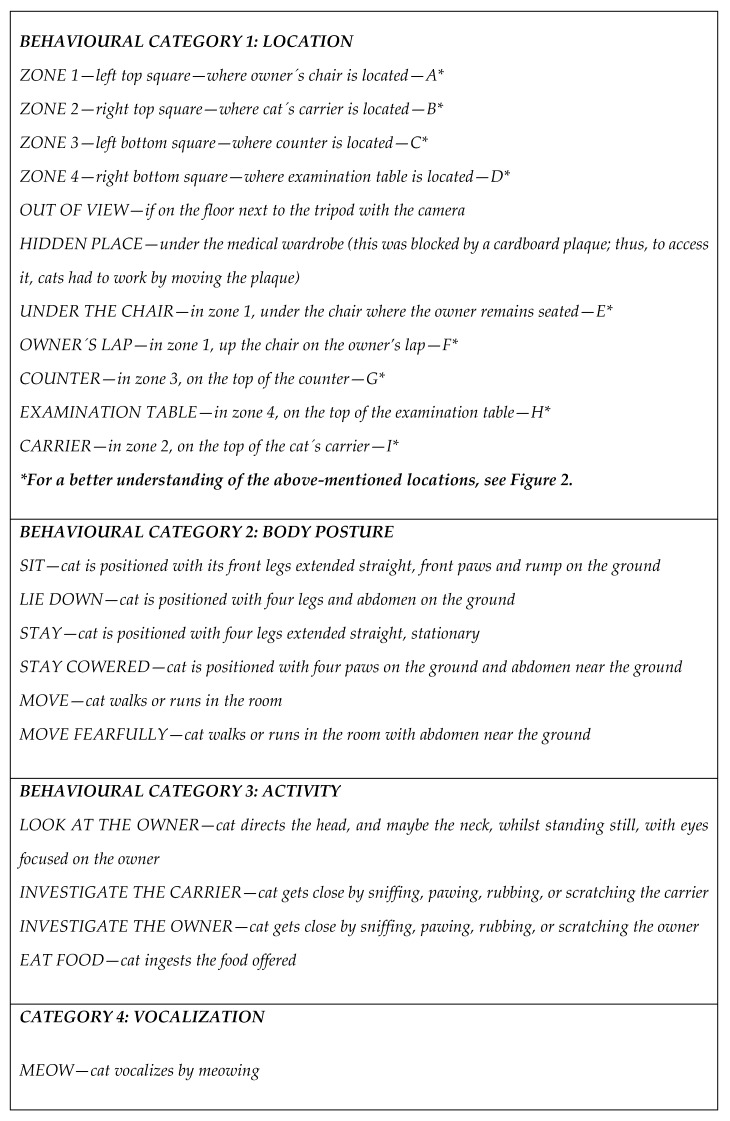
Behavioural categories and defined behaviours for video analysis.

**Figure 2:**
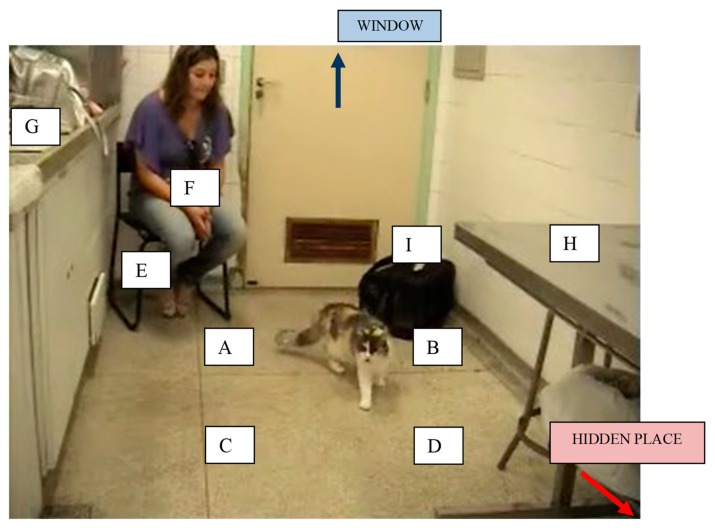
Dedicated room where the recording took place. Locations are identified by letters A–I. Hidden place: from a window at the door, it was possible to register the moment the cat went from out of view to the hidden place location. This was under a medical cabinet located in the room.

Behavioural categories and their definition (see Figure 1) were determined before video analysis started. Videos were analysed in terms of the cat’s location in the room (A, B, C, D, E, F, G, H, I, out of view, in the hidden place, see Figure 2), posture (stopped—sit, lie down, stand, cowered—moving normal, moving fearfully), activity (look at the owner, investigate the carrier, investigate the owner, eat food), and vocalization (meow). From a window in the door, it was possible to see the moment the cat went from out of view to the hidden place and thus one of the researchers recorded these times. Videos were evaluated by a “blind” observer who did not know any of the cats nor the purpose of the study. Coding was undertaken with the software Solomon Coder beta 11.04.23 (ELTE TTK, Department of Ethology, Budapest, Hungary).

### 2.4. Statistical Analysis

Shapiro–Wilk normality test was first performed for each of the aforementioned variables in both groups (“cases” and “controls” were first tested separately). In the case of normal distributions for both the “cases” and “controls”, Student paired *t*-test was selected for a comparison between them in that given group. Wilcoxon signed-rank test was chosen in the case of non-normal distributions in the tests (i.e., “cases”, “controls”, or both of them). In addition, groups S and L were compared as a unit, i.e., considering the participant cats together (i.e., “cases” + “controls”). For this, two sample *t*-test or Mann–Whitney test was used depending on the distribution of the variable: If a non-normal distribution was previously seen in at least one test (“cases”, “controls”, or both of them) in one of the groups, a non-parametric test was used. Statistical analysis was performed using SAS software (9.2 version, SAS, Cary, NC, USA) and a probability level of 0.05 was used to assess significance.

Several of the behavioural variables were rarely manifested by the cats and thus were not used for statistical comparisons but briefly described in both groups. “Case” and “control” cats were thus compared as a function of the duration of the following variables: At the hidden place, close to the owner (A, B, under the owner’s chair), far from the owner (C, D, out of view), stopped (sit + lie down + stand + stand cowered), moving, and attentive to the owner (looking owner + investigating owner). For “far from owner/(far + close) from owner” and “moving/(stopped + moving)”, proportions were calculated and then used for a comparison between “cases” and “controls”. Comparisons were also made between “cases” and “controls” as a function of the frequency of the following: Meow and attentive to the owner.

## 3. Results

### 3.1. Faecal Cortisol Metabolites (FCMs)

#### 3.1.1. Group S—Spraying Dyads

The owner of one dyad was unable to collect faeces of the cats as she never witnessed defecation of the participant cats. The results of the remaining 10 “case-control” dyads are therefore presented.

**Figure 3:**
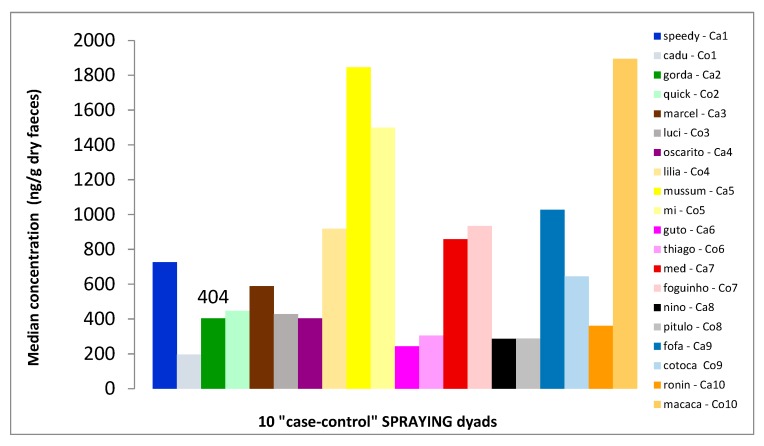
Faecal cortisol metabolites (FCMs) for the 10 “case-control” spraying dyads. “Ca” = case cat, “Co” = control cat.

Whilst “sprayer” cats had a median level of FCMs equal to 497 ng/g dry faeces (IQR = 497.75; n = 10), the median level of their “controls” was 545 ng/g dry faeces (IQR = 325; n = 10). One “case” cat (Mussum), and one “control” cat (Macaca) had levels clearly different from the rest of the group (i.e., Mussum = 1846 ng/g dry faeces; Macaca = 1894 ng/g dry faeces) and thus could be considered as potential outliers (Figure 3). However, given the large variation in FCM levels, and the fact that other cats approximated to the outliers in their values, we decided to keep them for the following inferential analysis. FCMs did not show significant differences when “case” and “control” cats were compared (Wilcoxon signed-rank test, *p* = 1.000).

#### 3.1.2. Group L—Latrining Dyads

**Figure 4:**
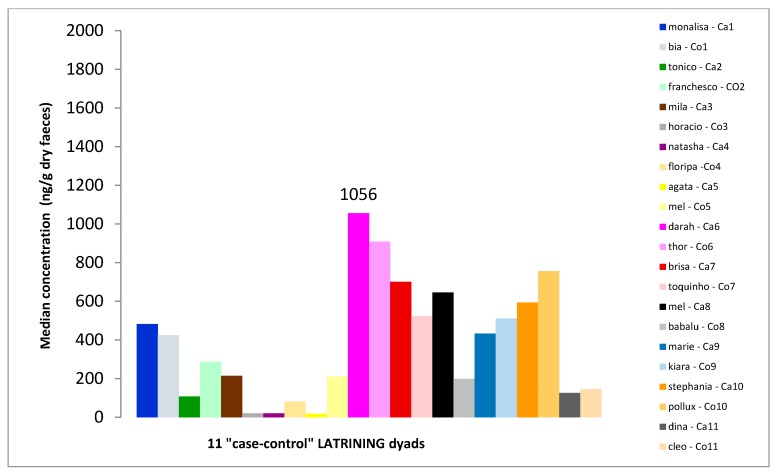
Faecal cortisol metabolites (FCMs) for the 11 “case-control” latrining dyads. “Ca” = case cat, “Co” = control cat.

The owner of one dyad was unable to collect faeces of the cats as she never witnessed defecation of the participant cats. The results of the remaining 11 “case-control” dyads are therefore presented. While “latriner” cats had a mean level of FCMs equal to 399 ng/g dry faeces (SD = 333; n = 11), the mean level of their controls was 369 ng/g dry faeces (SD = 283; n = 11) (Figure 4). FCM levels of “case” and “control” cats did not show significant differences (Student paired *t*-test, *p* = 0.625).

#### 3.1.3. Group S X Group L

FCM levels in group S (spraying = “sprayers” + controls) were significantly higher than group L (latrining = “latriners” + controls) (Mann–Whitney test, *p* = 0.026).

### 3.2. Behaviours Recorded

#### 3.2.1. Group S—Spraying Dyads

An analysis of videos from 18 cats (i.e., nine dyads) was performed. Videos from the three remaining dyads had to be excluded due to the owner’s interference (e.g., picking up the cat onto the lap, etc.), despite clear instructions not to do so.

Most cats spent a great proportion of the time in the hidden place (“cases”—median = 131.60 s, IQR = 89.00; “controls”—median = 108.40 s, IQR = 133.20). There was no significant difference between “cases” and “controls” in either the time spent hidden (Wilcoxon signed-rank test, *p* = 0.843) or in close proximity with the owner (median of 2.20 s (IQR = 31.40) versus 8.40 s (IQR = 51.00); Wilcoxon signed-rank test, *p* = 0.4609) and far from the owner (i.e., mean of 43.20 s (IQR = 4.00) versus 41.40 s (IQR = 16.00); Wilcoxon signed-rank test, *p* = 0.480).

None of the cats stayed on the top of its carrier (i.e., location I) nor on the owner’s lap (i.e., location F). While only one “case” cat jumped onto the table (i.e., location H) and none *into the sink* (i.e., location G), only one “control” cat jumped into the sink and none onto the table.

Concerning the proportion of time spent far from owner/(far + close) from owner, “case” cats did not significantly differ to the “controls” (“cases”—median was 4.68%, IQR = 38.51; “controls”—median was 17.14%, IQR = 49.92, Wilcoxon signed rank test, *p* = 0.425), thus indicating that “cases” and “controls” spent a similar time distant (proportionally to close) from the owner.

As for looking at the owner and investigating the owner, when the two were combined, there was also no significant difference between “cases” and “controls” (Wilcoxon signed-rank test, *p* = 0.578). While “cases” spent about 1.40 s (IQR = 16.60), “controls” spent about 0.00 s (IQR = 1.00) on this composite behaviour. Significant differences were also not found when looking at the owner plus investigating the owner behaviour was considered in terms of its frequency (“cases”: median = 1.00 times, IQR = 3.00; “controls”: median = 0.00 times, IQR = 1.00; Wilcoxon signed-rank test, *p* = 0.703).

As to meowing, samples were small: Spraying cats meowed a median of 7.00 times (IQR = 26.00, n = 6), their “controls” meowed a median of 0.00 times (IQR = 9.00, n = 4). There was no significant difference in terms of thee frequency (Wilcoxon signed-rank test, *p* = 0.437).

Concerning time spent stopped versus moving, while spraying cats spent on average 5.80 s (IQR = 32.00) moving and 1.00 s (IQR = 3.40) stopped (always in a standing posture), their “controls” spent on average 2.00 s (IQR = 18.80) moving and 0.40 s (IQR = 90.40) stopped (in sit, lie down, cower, or standing postures) (Wilcoxon signed-rank test, *p* = 0.359 (moving) and *p* = 0.375 (stopped)). About the move/(move + stop) proportion, “cases” and “controls” did not significantly differ: Thee “cases” median was 87.88%, IQR = 28.17 s; the “controls” median was 64.32%, IQR = 77.45, Wilcoxon signed-rank test, *p* = 0.312), thus indicating that, “cases” and “controls” spent a similar time moving (proportionally to stopped) around the room.

Three “case” and two “control” cats ate the food provided.

#### 3.2.2. Group L—Latrining Dyads

An analysis of videos from 16 cats (i.e., eight dyads) was performed. Videos from the four remaining dyads had to be excluded due to owner interference, at least with one of the cats (e.g., taking the cat out of the hidden place, etc.), despite clear instructions not to do so.

Most cats spent a great proportion of the time in the hidden place (“cases”—median = 12.70 s, IQR = 106.80; “controls”—median = 68.70 s, IQR = 92.80). There were no significant differences between “cases” and “controls” as to time spent neither hidden (Wilcoxon signed-rank test, *p* = 0.382) nor in close proximity with the owner (i.e., median = 50.40 s (IQR = 152.60) versus 37.20 s (IQR = 76.8); Wilcoxon signed-rank test, *p* = 0.312) or far from the owner (i.e., mean = 50.70 s (SD = 46.41) versus 52.48 s (DP = 28.68); Student paired *t*-test, *p* = 0.916).

None of the cats stayed on the top of its carrier (i.e., location I) and two (i.e., one “case” and one “control”) stayed on the owner’s lap (i.e., location F). While only one “case” cat jumped onto the sink (i.e., location G) and none onto the table (i.e., location H), “control” cats did not jump either into the sink or onto the table.

Concerning the proportion of time spent far from the owner/(far + close) from the owner, “case” cats did not significantly differ to the “controls” (“cases”—mean was 50.77%, SD = 40.12; “controls”—mean was 37.85%, SD = 33.82, Student paired *t*-test *p* = 0.566).

As to the combination of looking at the owner and investigating the owner behaviours, there was also no significant differences between “cases” and “controls” (Wilcoxon signed-rank test, *p* = 0.812). While “cases” spent a median of 4.20 s (IQR = 11.20), “controls” spent a median of 0.90 s (IQR = 13.20) on this composite behaviour. No significant difference was found when the frequency of looking at the owner plus investigating the owner behaviour was considered as a single measure (“cases”: median = 3.00 times, IQR = 5.00; “controls”: median = 1.00 times, IQR = 5.50; Wilcoxon signed-rank test, *p* = 0.656).

As to the meowing frequency, “cases” and “controls” did not significantly differ. While inappropriate toileting cats meowed a median of 3.50 times (IQR = 16.00), their “controls” meowed a median of 0.50 times (IQR = 10.00) (Wilcoxon signed-rank test, *p* = 0.437).

Concerning time spent stopped versus in movement, while latrining cats spent a median of 8.10 s (IQR = 10.00) moving and 73.15 s (DP = 63.80) stopped (in sitting, cowered, and\or standing posture), their “controls” spent a median of 15.00 s (IQR = 22.20) moving and 36.38 s (DP = 38.10) stopped (in sitting, cowering, and\or standing postures) (Student paired *t*-test, *p* = 0.262 (stopped); Wilcoxon signed-rank test, *p* = 0.546 (moving)). About the move/(move + stop) proportion, “cases” and “controls” did not significantly differ: The “cases” median was 15.18%, IQR= 47.85; while the “controls” median was 50.06%, IQR = 49.54, Wilcoxon signed-rank test, *p* = 0.156), thus indicating that “cases” and “controls” spent a similar time moving (proportionally to stopped) around the room.

Two “case” and one “control” cat ate the food provided.

#### 3.2.3. Group S X Group L

When all cats in each group were considered together in terms of the behaviours recorded, and an in-between subjects comparison was made, and differences arose. When looking at move\(move + stop) proportions, cats from spraying houses spent significantly more time moving (in proportion to stop) (Mann–Whitney test, *p* = 0.011). When looking at time spent stopped (duration), cats from latrining houses spent a longer time than cats from spraying houses (Mann–Whitney, *p* = 0.050).

## 4. Discussion

Considering faecal cortisol metabolites (FCMs) as a physiological parameter of arousal underpinning emotional stress, evidence for the hypothesis of spraying being a feline stress reaction still remains to be demonstrated, despite earlier claims [19]. Comparisons made between spraying and latrining households both in terms of FCMs and behaviours recorded suggest all cats from the *spraying* households were more aroused overall but not all reacted with spraying.

The question which then arises is: “If equally aroused, why do only some exhibit urine spraying?” The answer to this question is not obvious and gives rise to several hypotheses. Spraying could be a sign of stress adaptation; in other words, it could represent an animal that has found a way to deal with a stressful condition. For instance, it might be that glucocorticoids levels in spraying cats could be even higher before spraying started, with a decrease happening to the currently seen levels. However, considering that these cats were engaged in the behaviour for so long, this hypothesis seems unlikely (a greater decrease would be expected if spraying was a successful strategy). Given that faecal glucocorticoids levels were significantly higher in cats from spraying houses (besides their significantly more active behaviours) in comparison with those from latrining houses, it is likely that “sprayers” were still as stressed as their controls. Thus, spraying by some cats may instead reflect an unsuccessful attempt to gain relief in a generally stressful environment. Further evidence for this hypothesis comes from a comparison with previously published research on laboratory cats whose faecal glucocorticoid metabolite levels (measured by the same 11-oxoaeticholanolone EIA-) were far lower than in this study [24,25]. For instance, while their median baseline level was 196 ng/g [25], ours was 518 ng/g (values considering both “case” and “control” cats). Indeed, some of the cats from our spraying households reached metabolite levels seen in cats after ACTH (adrenocorticotropic hormone) injection [25]. In a further study [26], faecal glucocorticoid metabolites from 57 domestic-owned cats coming from multi-cat houses of three to four cats were also measured. Mean FCM levels were 227 ng/g. It is important to note that none of the owners, in this latter study, mentioned spraying as a behaviour manifested by any of their cats and indeed overt behavioural problems, such as severe aggression, were considered as an exclusion criterion.

As it stands, spraying behaviour seems to arise in a generally high arousal (probably stressful) environment and does not seem to be associated with a calming effect, as suggested by some [10]. As glucocorticoid concentrations of both our “sprayers” and “controls” were probably high, the best explanation seems to be that spraying might function as a coping strategy [27,28] that seems to be associated with higher arousal, and indeed it might be that the spraying helps to maintain this heightened state. The spray from a single cat may be sufficient to elicit this state among all household members. Territoriality and competing for mates (both of which are well-established contexts for spray marking) may be framed as attempts to hold a resource against a threat, which would be associated with the elicitation of emotional frustration [29], which is characterised by high arousal. By contrast, hiding behaviour by domestic cats [30] is associated with a clear decrease in excreted urinary cortisol by caged laboratory cats that hid behind the litter tray when under a stressful condition [30]. The lack of significant differences between “sprayers” and “controls” in the behaviours seen in the videos seem to support our conclusion that “sprayers” do not differ in their level of arousal. The more active type of behaviour manifested by cats from “spraying” households overall is also consistent with a higher arousal state compared to those from households with a latrine issue. Nonetheless, given the recorded faecal glucocorticoid values from the aforementioned studies, it seems that even those from latrining households might be living in stressful environments but to a lesser degree than those from spraying households. It is possible, however, that the number of cats is also a feature here. Although there were households of three to nine cats in both groups, spraying households had significantly more cats than latrining ones (two sample *t*-test, *p* = 0.011).

The importance of outside access on arousal levels is questionable. It was available within five spraying and none of the latrining houses. A lack of outside access has previously been reported to be associated with both latrine-related issues [13] and access may be related to the risk of high arousal problem behaviours, such as aggression [31,32]. Higher arousal could also, at least in part, be explained by other factors not investigated in this research (e.g., owner attitudes towards the cats, cat group composition, time living together, kinship, time when last cat was incorporated, etc.). Although additional studies are required to determine the effects of spraying on cats already aroused, the present results support the recommendation that the use of aversive techniques for spraying behaviour (e.g., punishment) even as a complementary technique as recommended in some textbooks (e.g., [33]) should not be part of the treatment plan, as this is likely to exacerbate any issues with arousal. Furthermore, it supports the need of appropriate management aimed at alleviating the strain from relevant stressors to be applied to the whole spraying household rather than just the “sprayer”. As it stands from these results, together with previous findings from the other part of this study [15], while latrining households seem to be high-risk “sick” environments with high levels of medical problems overall, spraying households seem to constitute highly aroused “stressful” homes for the cats.
